# *Listeria monocytogenes* Strains Persisting in a Meat Processing Plant in Central Italy: Use of Whole Genome Sequencing and In Vitro Adhesion and Invasion Assays to Decipher Their Virulence Potential

**DOI:** 10.3390/microorganisms11071659

**Published:** 2023-06-26

**Authors:** Giuditta Fiorella Schiavano, Fabrizia Guidi, Francesco Pomilio, Giorgio Brandi, Romolo Salini, Giulia Amagliani, Gabriella Centorotola, Francesco Palma, Martina Felici, Cinzia Lorenzetti, Giuliana Blasi

**Affiliations:** 1 Dipartimento di Studi Umanistici, Università degli Studi di Urbino “Carlo Bo”, Via Bramante, 17, 61029 Urbino, Italy; 2 Istituto Zooprofilattico Sperimentale dell’Abruzzo e del Molise G. Caporale, Laboratorio Nazionale di Riferimento per Listeria Monocytogenes, Via Campo Boario, 64100 Teramo, Italy; f.guidi@izs.it (F.G.);; 3 Dipartimento di Scienze Biomolecolari, Università degli Studi di Urbino “Carlo Bo”, Via Santa Chiara, 27, 61029 Urbino, Italy; 4 Istituto Zooprofilattico Sperimentale dell’Abruzzo e del Molise G. Caporale, Centro Operativo Veterinario per l’Epidemiologia, Programmazione, Informazione e Analisi del Rischio (COVEPI), National Reference Center for Veterinary Epidemiology, Via Campo Boario, 64100 Teramo, Italy; 5 Istituto Zooprofilattico Sperimentale dell’Umbria e delle Marche “Togo Rosati”, Via Gaetano Salvemini, 1, 06126 Perugia, Italy

**Keywords:** *Listeria monocytogenes*, whole genome sequencing (WGS), virulence profile, premature stop codon (PMSC), adhesion and invasion assays

## Abstract

In this study, we used both a WGS and an in vitro approach to study the virulence potential of nine *Listeria monocytogenes* (*Lm*) strains belonging to genetic clusters persisting in a meat processing plant in Central Italy. The studied clusters belonged to CC1-ST1, CC9-ST9, and CC218-ST2801. All the CC1 and CC218 strains presented the same accessory virulence genes (LIPI-3, gltA, gltB, and aut_IVb). CC1 and CC9 strains presented a gene profile similarity of 22.6% as well as CC9 and CC218 isolates. CC1 and CC218 showed a similarity of 45.2% of the same virulence profile. The hypervirulent strains of lineage I (CC1 and CC218) presented a greater ability to adhere and invade Caco-2 cells than hypovirulent ones (CC9). CC1 strains were significantly more adhesive and invasive compared with CC9 and CC218 strains, although these last CCs presented the same accessory virulence genes. No statistically significant difference was found comparing CC218 with CC9 strains. This study provided for the first time data on the in vitro adhesiveness and invasiveness of CC218-ST2801 and added more data on the virulence characteristics of CC1 and CC9. What we observed confirmed that the ability of *Lm* to adhere to and invade human cells in vitro is not always decipherable from its virulence gene profile.

## 1. Introduction

Listeriosis, caused by *Listeria monocytogenes (Lm),* is a severe disease recognised as the most serious foodborne infection under EU surveillance and presenting the highest proportion of hospitalized cases (96.5%) and fatality rates (13.7%) [1]. Over the last few years, foodborne outbreaks of listeriosis have been documented all over the world, across Asia, Africa, Europe, and America [2,3,4,5,6,7,8,9]. Infection by *Lm* results in either non-invasive gastrointestinal listeriosis or invasive listeriosis, which more frequently among risk categories such as immunocompromised people [10,11]. Invasive listeriosis can lead to abortion in pregnant women and cause septicaemia and meningitis in other patients [12]. Infection by *Lm* is often associated with consumption of contaminated ready-to-eat meat, dairy, and fishery products [13]. *Lm* is well-adapted to a wide range of conditions in food (i.e., along the food chain) and can persist within food processing environments (FPEs). This microorganism is able to growth in a wide range of stressful environmental conditions such as low and high temperatures (−2–45 °C), both acidic and basic pH (4.0–9.6), low water activity (aw ≥ 0.92), and high salinity (up to 12% *w*/*v*) [14]. The adaptation of this pathogen to the stress conditions present in the FPEs of meat and other foods, including cleaning and disinfection processes and food storage procedures, favours its persistence and increases the risk of food contamination [15,16]. The persistence of *Lm* in FPEs can last for years and represents one of the most concerning aspects of this pathogen, since it constitutes a risk to the health of the consumer. *Lm* is a highly heterogeneous species with regard to its virulence potential. It can be divided into four evolutionary lineages [17], four PCR serogroups, and 14 serotypes [18,19]. *Lm* isolates can be grouped into more than 3000 sequence types (STs) that group in different clonal complexes (CCs) [20]. It is well-known that *Lm* clones that predominate in clinical cases and in food samples have different occurrences, and there is evidence for a high variability of pathogenicity and stress response of different CCs. In particular, certain CCs such as CC1, CC2, CC4, and CC6 are more frequently associated with clinical cases and are hypervirulent in a humanised mouse model, whereas others, such as CC9 and CC121 of lineage II, are mainly linked to a food origin, present a better adaptation to environmental stresses, and show hypovirulence in vivo [21,22,23,24,25,26,27].

*Lm* is an intracellular pathogen with a complex life cycle that allows bacteria to spread in tissues while remaining in the cytosol of host cells [12,21]. This life cycle is thought to protect *Lm* from the humoral immune response [28]. Upon ingestion of contaminated food by the host, *Lm* reaches the stomach, where it faces an extremely acidic pH, the first physicochemical antimicrobial host barrier, followed by other gastrointestinal tract defence mechanisms (i.e., peristalsis and the mucus-layer lining of the gastrointestinal tract) [29]. *Lm* has the ability to actively induce its internalization into non-phagocytic cells, including intestinal epithelial cells, hepatocytes, and placental trophoblast cells [12,28]. The virulence factors of *Lm* are either scattered across the genome (e.g., the *inlA–inlB* locus, *bsh*, *inlC*, and *lap*, among others) or clustered in pathogenicity islands such as Listeria Pathogenicity Island 1 (LIPI-1), LIPI-3, and LIPI-4. The *inlA–inlB* locus encodes two surface proteins, internalins InlA and InlB, involved in pathogen internalization by non-phagocytic cells after binding of the host cell receptors E-cadherin and Met, respectively. The *inlA–inlB* locus, together with LIPI-1 (*actA*, *prfA*, *hlyA*, *mpl*, *plcA*, and *plcB*), is necessary for key steps of intracellular parasitism (e.g., adhesion, internalization, intracellular survival, and dissemination). Importantly, the *inlA–inlB* locus and LIPI-1 are conserved in almost all *Lm* isolates, highlighting their crucial role for pathogenicity [16]. Listeriolysin S (LLS) encoded by LIPI-3 (*llsA*, *llG*, *llsH*, *llsX*, *llsB*, *llsY*, *llsD*, and *llsP*) is a haemolytic cytotoxic factor associated with the destruction of gut microbiota and thus dysregulates host–microbiota homeostasis during infection [30]. More recently, a cluster of six genes (*GlvA, Gat-pr, YdjC, GatA, GatB,* and *GatC*) encoding a cellobiose-family phosphotransferase system (PTS) were identified as LIPI-4 [22]. This PTS has been identified as a virulence factor implicated in maternal–neonatal and central nervous system infections. Both LIPI-3 and LIPI-4 genes are considered hypervirulent factors strongly associated with *Lm* invasive infections [18]. Genome analyses have confirmed the diversity of virulence factors among hypo- and hypervirulent clones and the presence of accessory virulence genes in the latter. Many studies using in vitro and in vivo approaches have been previously conducted in order to better understand the pathogenesis of *Lm.* In particular, its ability to adhere to and invade phagocytic and non-phagocytic cells represents a key aspect which consists of multiple stages, including cell adhesion, internalisation, vacuolar escape, intracellular replication, movement by actin mobilisation, and cell-to-cell spread [31]. The wide range of virulence factors involved in *Lm* pathogenesis and the related encoding genes have been identified, at first, throughout classical genetics and later with the advent of whole genome sequencing (WGS), which opened a new avenue for increasing our understanding of genetic virulence [32]. Multiple regulation mechanisms control the expression of virulence determinants in *Lm*, and some of them are also connected with environmental stress responses [33,34,35]. In this study, we used an integrated approach combining WGS and in vitro assays to study the virulence of *Lm* strains belonging to persistent clusters, detected from nonfood contact surfaces in a single meat processing plant in Central Italy during an extensive environmental sampling carried out between July 2020 and September 2021. Our double goal was to evaluate the virulence gene profile of different *Lm* clones persisting in the same FPE and to verify whether the in vitro adhesion and invasion abilities of these strains were predicted from the presence/absence of specific virulence genes. Particular attention was paid to *Lm* strains identified with the new MLST profile ST2801 (CC218) and belonging to the atypical serogroup IVb-v1. No data on the in vitro adhesion and invasion abilities and their relationship to the virulence gene profile are currently available for this new ST.

## 2. Materials and Methods

### 2.1. Bacterial Isolates

The nine *Lm* strains used in this study were isolated in a meat processing plant in Central Italy during an extensive environmental sampling for *Lm.* The meat processing plant was specialised in pork meat cutting and production of different meat products and sausages typical of Central Italy. In the period between July 2020 and September 2021, three different sampling sessions were performed during production. A sampling scheme of 63 surfaces, including both food and nonfood contact surfaces was defined, focusing on the main niches for *Lm* presence. In each sampling session, the same surfaces were sampled using commercial sterile sponges. In accordance with the European Union Reference Laboratory for *Lm* (EURL) guidelines [36], the total sampled area varied depending on the sampling site but was as large as possible to improve the probability of detecting *Lm*. The samples were tested according to ISO 11290-1:2017 for *Lm* detection.

All the strains of this study were isolated from nonfood contact surfaces sampled during production and belonged to genomic clusters found to persist inside the plant. In more detail, two strains belonged to CC1-ST1 (serogroup IVb), four strains belong to CC9-ST9 (serogroup IIc), and the remaining three strains belonged to CC218-ST2801 and presented the atypical IVb-v1 serogroup profile [37]. CC1 and CC218 strains were selected for this study as they belonged to hypervirulent CCs, while CC9 strains were representatives of a hypovirulent CC.

According to the cgMLST analysis, using a threshold of seven allelic distances for cluster definition [38], the CC1 strains were representative of the same cluster persisting from July 2020 to September 2021. The CC9 strains grouped into the same cluster repeatedly isolated from July 2020 to September 2021 both during production and after two consecutive cleaning and sanitation sessions. The three CC218 isolates were representative of three genetic clusters, genetically close each other. Two of these clusters (Clusters A and B) persisted from July 2020 to September 2021, both during production and after cleaning and sanitation sessions. The remaining cluster (Cluster C) was detected from September 2020 to May 2021 and only during production.

Data on the *Lm* strains used for the study are summarised in Table 1. More details are reported in Table S1 (Supplementary Materials).

### 2.2. Whole Genome Sequencing (WGS) and Bioinformatic Analysis

#### 2.2.1. Next Generation Sequencing and Genome Assembly

DNA extraction was performed using a QIAamp DNA Mini Kit (Qiagen Hilden, Germany) according to the manufacturer’s protocol with minor modifications according to Portmann et al. 2018 [39]. DNA quantity and quality were evaluated with a Qubit fluorometer (Thermo Fisher Scientific, Waltham, MA, USA) and Eppendorf BioSpectrometer fluorescence (Eppendorf s.r.l., Milano, Italy). DNA integrity was assessed with an Agilent 4200 TapeStation system (Agilent, Santa Clara, CA, USA). Starting from 1 ng of input DNA, the Nextera XT DNA chemistry (Illumina, San Diego, CA, USA) for library preparation was used according to the manufacturer’s protocols. WGS was performed on the NextSeq 500 platform (Illumina, San Diego, CA, USA) with the NextSeq 500/550 mid-output reagent cartridge v2 (300 cycles, standard 150 bp paired-end reads). For the analysis of WGS data, an in-house pipeline [40] was used which included steps for trimming (Trimmomatic v0.36) [41] and a quality-control check of the reads (FastQC v0.11.5) [42]. Genome de novo assembly of paired-end reads was performed using SPAdes v3.11.1 [43] with default parameters for the Illumina platform 2 × 150 chemistry (--only-assembler, --careful, and --k21, 33, 55, and 77). Subsequently, the genome assembly quality check was performed with QUAST v.4.3 [44]. All the genomes that met the quality parameters recommended by Timme et al. 2020 [45] were used for the analysis. The genome assemblies were deposited at DDBJ/ENA/GenBank under the BioProjects PRJNA961709 (Lm_2529, Lm_2604, Lm_2527, Lm_2603, Lm_2516, and 2021.TE.312882.1.40) and PRJNA821663 (Lm_2513, Lm_2525, and Lm_2568) (Supplementary Materials Table S1).

#### 2.2.2. Multilocus Sequence Typing Analysis

A multilocus sequence typing (MLST) analysis was performed, deducting in silico the ST and CC by using the specific tool available in the BIGSdb-*Lm* database (https://bigsdb.pasteur.fr/listeria/; accessed on 1 February 2023) [20].

#### 2.2.3. Genetic Determinants Involved in Virulence Potential

All the genome assemblies were manually screened for the presence/absence of loci encoding for *Lm* virulence using the “Virulence” tool provided by the BIGSdb-*Lm* platform, and also investigating the presence of premature stop codon mutations (PMSC) in the *inlA* gene (accessed on February 2023).

### 2.3. In Vitro Adhesion and Invasion of Lm Isolates in Caco2 Cells

#### 2.3.1. Epithelial Cell Line

Human colon carcinoma epithelial cell line (Caco-2) (ECACC 86010202) cells were obtained from the European Collection of Authenticated Cell Culture (St. Louis, MO, USA). Caco-2 cells were cultured as monolayers in 75 cm^2^ flasks with Dulbecco’s modified Eagle’s medium (DMEM) containing 10% (vol/vol) heat-inactivated foetal bovine serum (FBS), 1% non-essential amino acids, 1% antibiotic solution (100 U/mL penicillin and 100 g/mL streptomycin), 1% L-glutamine, and 1% sodium pyruvate. Once the flasks reached 90% confluence, the cells were digested using trypsin and seeded at a desirable density onto 6-well plates (Corning Inc., New York, NY, USA). Plates were incubated at 37 °C and 5% CO_2_ for at least 24 h to achieve full confluence after seeding. All cell culture materials were purchased from Sigma-Aldrich (St. Louis, MO, USA).

#### 2.3.2. Adhesion Assay

Two days prior to the adhesion assay, Caco-2 cells were seeded in 6-well plates to obtain semiconfluent monolayers (1.5 × 10^5^ cells/mL), as described by Schiavano et al. 2022 [46]. On the day of the assay, cells were washed with phosphate-buffered saline (PBS, pH 7.4), and fresh prewarmed medium without FBS was added to the wells. Bacteria were grown in TSYEB to the mid-logarithmic growth phase at 37 °C with shaking at 200 rpm and used in the experiment measured and adjusted to an OD_600nm_ = 0.1. Bacterial number was confirmed by plating ten-fold dilutions of the bacterial suspension onto TSYEA and incubated at 37 °C for 24 h. After incubation, the bacterial concentration of the culture was determined by the number of colony-forming units (CFU) by plating serial dilutions on TSYEA. Plates were incubated at 37 °C for 24 h. The Caco-2 cells grown in 6-well tissue culture plates were infected with 10^7^ bacteria to yield a multiplicity of infection (MOI) of approximately 100 CFU per cell. The precise number of inoculated bacterial CFU added at time zero (T_0_) was determined by plating the serial dilution on TSYEA. To synchronise adhesion without forcing adhesion, bacteria were spun down on the cell layer for 1 min at 200× *g*. After incubation at 37 °C and 5% CO_2_ for 1 h with bacteria to allow adherence, the monolayers were thoroughly washed five times in cold PBS to remove the bacteria that had not adhered. Serial dilutions were plated on TSYEA and incubated at 37 °C for 24 h; then, the *Lm* colonies were enumerated to determine the number of adhered bacteria. The adhesion efficiency (%) for each strain was expressed as the percentage of the number of bacteria attached to the cells compared with the total number of CFU provided in the inoculation multiplied by 100. Noninfected wells were used as negative controls, and each assay was performed with triplicate samples for each strain. *L. innocua* ATCC33090 was included as a negative control.

#### 2.3.3. Invasion Assay

Caco-2 cells were infected as described in Section 2.3.2 and incubated at 37 °C and 5% CO_2_ [46]. After 3 h post-infection, cells were washed five times with cold PBS, and fresh medium containing 50 µg/mL gentamycin (Sigma Aldrich, St. Louis, MO, USA) was added for an additional 90 min of incubation under the same conditions to kill the extracellular bacteria. After incubation, cells were extensively washed with cold PBS to remove gentamycin; then, the intracellular bacteria were recovered by lysis of the monolayers using 500 µL of cold 0.1% Triton X-100 (Merck) and sonication (Fisher Scientific Sonic Dismembrator Model 100, Pittsburgh, PA, USA, setting 3, three pulses, 6 s each). The resulting suspension was diluted 10-fold, spread on TSYEA, and incubated at 37 °C for 24 h. The number of CFU was considered as the number of bacteria that had invaded the Caco-2 cells; it was considered that the counts obtained 3 h after the onset of infection represented the number of bacteria that had been internalised. Uninoculated wells were used as negative controls, and each infection was carried out in three replicates for each strain. *L. innocua* ATCC33090 was included as a negative control. The invasion level (%) of each strain was calculated by dividing the number of CFU that invaded the cells after 90 min of incubation in gentamicin-containing medium by the total number of CFU obtained without gentamycin treatment and was expressed as a percentage.

### 2.4. Statistical Analysis

Mean value and standard deviation of adhesion and invasion levels were calculated from biological replicates performed in triplicate. An unpaired, two-tailed *t*-test was applied to evaluate the statistical differences between the adherent bacteria or intracellular bacteria and the reference negative control (*L. innocua* ATCC 33090). *p*-values < 0.05 were considered to be significant. The analyses were conducted using GraphPad Prism8 Software (GraphPad Software Inc., San Diego, CA, USA). For both adhesiveness and invasiveness, an ANOVA was applied to test for overall differences between the averages followed by multiple comparisons with *t*-test and Bonferroni correction. We extended the analysis by comparing CCs with each other (e.g., CC1 vs. CC9; CC1 vs. CC218; and CC218 vs. CC9) and comparing all hypervirulent vs. hypovirulent CCs. The statistical analyses were performed using R (version 4.0.2, R Foundation for Statistical Computing, Vienna, Austria).

## 3. Results

### 3.1. Whole Genome Sequencing (WGS) Data Analysis

All the genomes met the quality parameters recommended and were used for the subsequent analysis steps. Using the BIGSdb-*Lm* platform, a total of 84 virulence genes were detected on a scheme of 93 targets (Supplementary Materials, Table S2). An amount of 55 genes were present in all the strains. Among these ubiquitous targets, there were the conventional LIPI-1, internalins genes including *inlA*, *inlB*, *inlC*, *inlC2*, *inlD*, *inlE*, *inlF, inlH*, *inlJ*, and *inlK*; *vip*; and the *viR*/*virS* virulence regulatory system. The variable genes showing differences in their presence/absence among different CCs were 18 (Supplementary Materials Table S3). Overall, the CC1 and CC218 strains presented 66 virulence genes, while CC9 isolates carried 60 targets. The virulence genes that were variable among the strains in terms of assigned allelic number were 72. Strains belonging to the same CC presented the same virulence profile both for presence/absence of virulence genes and for the relative allelic variant. In Table S2 (Supplementary Materials), we reported the dataset showing the allelic variant of all the virulence genes for each strain. In more detail, all the CC1 and CC218 strains presented the additional LIPI-3, the teichoic acid biosynthesis genes *gltA* and *gltB*, and the invasion gene *aut_IVb*. The virulence genes *ami*, *out*, and *tagB* were carried by all the isolates belonging to CC9, as well as the internalins genes *inlG, inlL*, and *LIPI2_inlII*. The CC9 isolates also presented a mutation in the *inlA* gene leading to a PMSC. In particular, in all the CC9 strains, the PMSC type 11 was identified consisting of a G-to-A substitution and leading to an InlA truncated protein of only 685 amino acids compared to the 800 amino acids of the wild-type protein. In *Lm* strains belonging to CC1 and CC218, a full-length *inlA* was found. Comparing the virulence profile between different CCs, CC1 and CC9 presented gene similarity of 22.6% (95% C.I.: 15.3–32.1%). The same result was observed for CC9 strains compared with those belonging to CC218. CC1 and CC218 strains showed 45.2% (95% C.I.:35.4–55.3%) of the same virulence profile (Table 2).

### 3.2. In Vitro Adhesion and Invasion of Lm Isolates in Caco2 Cells

**Figure 1:**
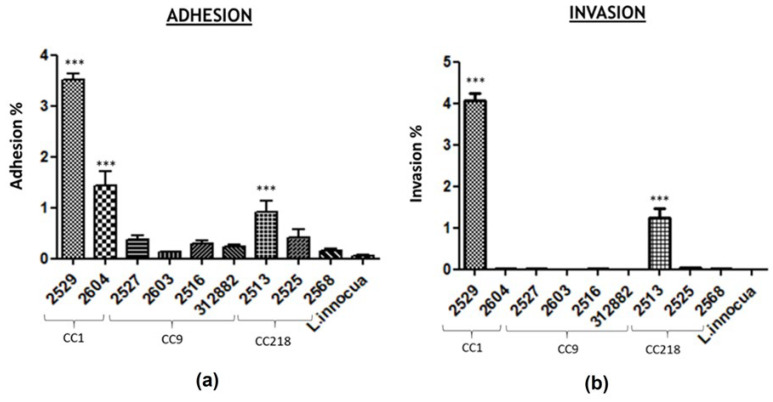
Caco-2 cell adhesion (**a**) and invasion (**b**) efficiency of *L. monocytogenes* isolates. Data were plotted as percentages of the starting viable inoculum. Data are the means ± standard deviation (SD) of three separate replicates for each strain. *p* < 0.05. The analyses were conducted using GraphPad Prism 8 software. * *p* < 0.05, ** *p* < 0.001, and *** *p* < 0.0001.

Human intestinal epithelial Caco2 cells were used to simulate in vitro the infection caused by *Lm*. The results of the adhesion and invasion efficiency in human intestinal epithelial cells of three CCs, each represented by two or more isolates, are reported in Figure 1.

This study included nine Lm strains of three persistent clusters belonging to CC1, CC9, and CC218, isolated in different sampling sessions from nonfood contact surfaces in the same meat processing plant. The strains belonged to serogroups IVb (two strains of CC1), IIc (four strains of CC9), and IVb-var1 (three strains of CC218). *L. innocua* ATCC 33,090 were included as a reference negative control. The adhesion level for all nine Lm strains ranged from 0.129% to 3.505%. For persistent cluster CC1, the highest levels of adhesion and invasion were detected compared to the isolates belonging to the other CCs. Lm_2529 and Lm_2604 showed adhesion levels of 3.505% (±0.31%) and 1.427% (±0.48%), respectively, which were significantly (*p* < 0.0001) higher than those of the negative control. The strains belonging to the CC9 cluster showed very low adhesion and invasion levels, ranging from 0.129% to 0.373% and from 0% (under detection limit) to 0.002%, respectively. The CC9 strain (Lm_2603) showed a percentage of invasiveness close to zero. The adhesion efficiency of the three CC218 strains ranged from 0.155% (±0.07%) for Lm_2568 to 0.910% (±0.37%) for Lm_2513, while the invasion levels ranged from 0.006% (±0.004%) for Lm_2568 to 1.240% (±0.22%) for Lm_2513, significant (*p* < 0.0001) with respect to the negative control only for Lm_2513.

### 3.3. Statistical Analysis

**Figure 2:**
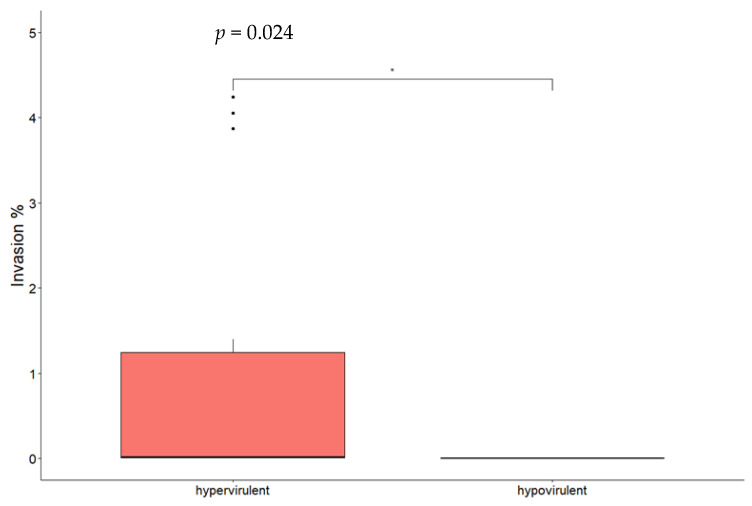
Box plot of *Lm* isolates’ adhesion efficiency to Caco-2 cells. Significance between hypervirulent and hypovirulent strains has been analysed (*t*-test and relative *p*-value in figure). * *p* < 0.05.

**Figure 3:**
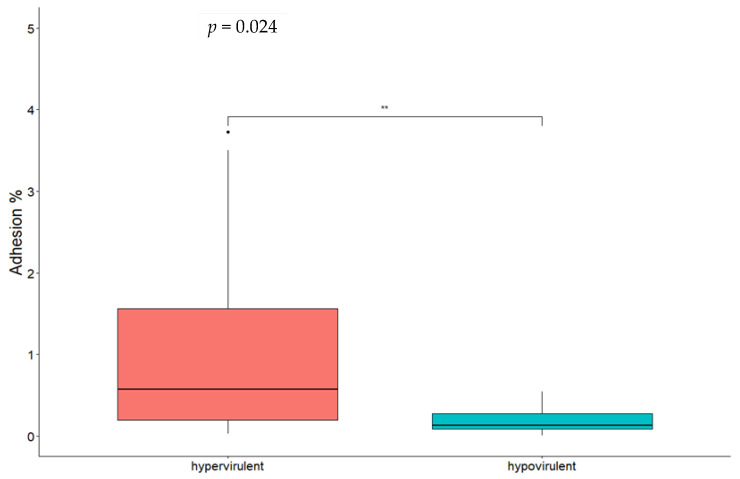
Box plot of *Lm* isolates’ invasion efficiency in Caco-2 cells. Significance between hypervirulent and hypovirulent strains has been analysed (*t*-test and relative *p*-value in figure). ** *p* < 0.001.

By comparing different CCs, significant differences for both adhesion (Figure 2) and invasion (Figure 3) were found between CC1 and CC9 strains and between CC1 and CC218 strains. No statistically significant difference was found comparing CC218 with CC9 strains. By comparing strains belonging to the same CC to each other, statistically significant differences in the adhesion ability were found between the two CC1 isolates and between CC218 strains Lm_2513 and Lm_2568. Significant differences in the invasion ability were found for the following pair comparisons: Lm_2529 vs. Lm_2604 (CC1), Lm_2527 vs. Lm_2603 (CC9), Lm_2603 vs. Lm_2516 (CC9), Lm_ 2513 vs. Lm_2525 (CC218), and Lm_2513 vs. Lm_2568 (CC218). Hypervirulent CCs (CC1 and CC218) were significantly more adherent and invasive compared with the hypovirulent CC9 strains (Figure 2 and Figure 3).

## 4. Discussion

The virulence potential of *Lm* strains persisting in a food processing plant represents a public health concern. Although any *Lm* strain can potentially cause disease in humans, especially in risk categories, the virulence assessment of strains repeatedly isolated in the environment of an FPE could provide useful information to evaluate the consumer’s risk.

In this study, we used both a WGS and an in vitro approach to study the virulence of nine *Lm* strains belonging to persistent clusters isolated in a meat processing plant in Central Italy during an extensive environmental sampling performed between July 2020 and September 2021.

Two strains belonged to CC1, one of the CCs commonly considered hypervirulent as presenting putative accessory virulence determinants and a high clinical frequency. Four strains belonged to CC9, considered hypovirulent and infecting immunocompromised people [22,32]. The remaining three strains belonged to CC218 and in particular to the ST2801, recently reported by Guidi et al. 2022 [37] for the first time and for which virulence potential was never studied before.

As reported by other authors, the virulence gene profiles were highly conserved within the individual CCs [38,47]. The virulence profile identified for each CC was the same reported by other authors in terms of genes presence/absence [38,47].

In more detail, CC1 strains carried a set of accessory determinants associated with increased virulence. One of them was the LIPI-3, a Listeriolysin S (LLS) biosynthetic cluster of eight genes, encoding for a haemolytic and cytotoxic factor required for *Lm* virulence in vivo. Other accessory genes presented by CC1 strains were *gltA* and *gltB*, involved in the expression of teichoic-acid-associated surface antigens in serogroup IVb. Moreover, all CC1 isolates carried a full-length *inlA* gene, encoding for a functional internalin A, considered one of the essential virulence factors for *Lm* to cross the intestinal barrier in order to invade epithelial cells [48,49]. The same accessory virulence genes were carried by CC218 strains with differences in allelic variants if compared with CC1 isolates.

On the other hand, CC9 strains also presented a set of accessory genes including *ami*, encoding an autolytic amidase involved in the adhesion to eukaryotic cells, the teichoic acid biosynthesis associated gene *tagB* and the internalin genes *inlG, inlL*, and *LIPI2_inlII.* As reported by other studies, the *inlG* and *inlL* were lacking in lineage I strains [50,51]. A recent study by Gou et al. 2022 [52] reported that the virulence of *Lm* strains during a mouse infection test decreased after the deletion of *inlG*, but the adhesion and invasion ability of these strains to Caco-2 cells was enhanced. The *inlL* gene encodes for a bacterial-surface-bound internalin (inlL) possessing a MucBP domain. The presence of a MucBP domain in InlL brought about the question of possible interactions of InlL with mucins, which also implies a role in bacterial adhesion [53]. It was demonstrated by Popowska et al. that InlL binds MUC2 but does not bind MUC1, already observed for InlB, InlC, and InlJ, but the study did not further investigate the significance of the MucBP domain for this interaction, leaving the issue unresolved [54]. Further studies are needed to better understand the detailed functional mechanism of *inlG* and whether it acts synergistically with other internalins. The specific role of the partial LIPI-2, specific for *L*. *ivanovii,* and in particular of the *inlII* gene in the virulence of these strains remains to be elucidated.

As reported in previous studies, all the CC9 and CC121 strains carried a PMSC mutation in the *inlA* gene, leading to a truncated protein [37]. In contrast, atypical hemolytic *L. innocua* strains harboring a functional inlA gene have been described [55].

As expected, within the same cluster, the virulence profile was also similar in terms of allelic variants of each gene. Comparing the different CCs, CC1 and CC218 strains presented the same accessory virulence genes with differences in allelic variants. The highest percentage of genes having the same allele between different CCs was found when comparing CC1 and CC218. The amount of genes that were equal between CC1 and CC9 was the same as that between CC218 and CC9. These results were expected and consistent with previous studies describing hypervirulent and hypovirulent *Lm* clones [37,38,46,47]. Once identified the virulence gene profile for each strain and compared it between different CCs; Caco-2 cell line cells were used to evaluate their effective adhesion and invasion abilities and to investigate if the virulence gene profile of these *Lm* strains may predict their virulence in vitro. Previously, other authors performed similar in vitro studies on CC1 and CC9 strains [37,38,39,40,41,42,43,44,45,46] but no data are available for the CC218-ST280.

The statistical analysis showed that when comparing all the hypervirulent strains (CC1 and CC218) with the hypovirulent ones (CC9), the hypervirulent ones presented a greater ability to adhere and invade Caco-2 cells, and this difference was statistically significant. By comparing different CCs, we found that CC1 was significantly more adhesive and invasive if compared with CC9. The same result was obtained comparing CC1 strains with CC218, while no statistically significant difference was found between CC218 and CC9. The first result was expected on the basis of the virulence gene profile, while the other ones indicated that although CC218 strains presented the same accessory virulence genes carried by CC1, they did not show similar behaviour in vitro, showing adhesion and invasion capabilities not significantly different from CC9 isolates.

Considering the strains individually, we observed that the CC1 Lm_2604 and the CC218 strains Lm_2525 and Lm_2568 presented a very low level of invasiveness despite carrying a full-length *inlA* gene and a complete LIPI-3. The highest levels of adhesion and invasion were showed by the CC1 strain Lm_2529. For this strain, the in vitro phenotype was consistent with its hypervirulent genetic profile. Similarly, the lower levels of adhesion and invasion showed by all the CC9 strains were explained by the presence of a PMSC mutation in the *inlA* gene. Several studies described the hypovirulence derived from this genotype in different model systems [22,38,46].

Since this study was the first to evaluate the in vitro virulence of the new ST2801, there are no previous data with which to compare our results. We tried to explain the unexpected results from the scientific literature available for different CCs. For example, Wagner et al. 2022 [47] similarly observed that the presence of LIPI-3 in CC3 strains of lineage I did not result in an increased in vitro virulence potential in human intestinal epithelial cells. This was explained by reporting that the encoded Listeriolysin S is only highly expressed in vivo in murine models [56]. In the same study, the CC14 strains of lineage II surprisingly showed a non-virulent phenotype despite the presence of a full-length *inlA* gene. The ensuing gene expression analysis performed by these authors detected a significantly lower *inlA* and *inlB* gene expression compared to the *Lm* EGDe strain as a reference, possibly due to a point mutation in the promoter region. With this in mind, as a future prospect, it should be useful to perform a gene expression analysis to understand whether the unexpected phenotype of our strains could be due to some regulatory mechanisms. Moreover, since several studies reported a mismatch between in vivo and in vitro results [52], it could be interesting to evaluate the virulence of the studied strains also using an in vivo model. For *Lm,* virulence is frequently assessed using a murine model [57]; however, the infection model of the greater wax moth *Galleria mellonella* has been employed in various studies as an alternative animal model and presents the advantages of cost efficacy, ease of manipulation, convenient growth conditions, and ethical acceptability [58].

Furthermore, Taveres et al. 2020 [59] reported that, differently from ST1 (CC1), ST218 (CC218) presented several non-synonymous single-nucleotide polymorphisms (SNPs) throughout LIPI-3 if compared with the wild form. However, it remains unclear if these point mutations in LIPI-3 have a significant impact in the *Lm* virulence. We did not verify the presence of these single-nucleotide polymorphisms throughout LIPI-3 in our CC218 strains; this is another interesting future prospect. However, in this study, the strains belonging to the same cluster were isolated from different surfaces and in different years; therefore, the variability in their in vitro virulence abilities could be due to the adaptation to different kinds of stresses. To date, it is still unknown whether adaptation to stress encountered in food or FPEs affects the pathogenicity of *Lm*. What is known is that exposure to stress conditions prompts a response leading to phenotypic changes that may remain even after the stress disappears.

More strains belonging to the same cluster should be tested to verify the inconsistent result. Moreover, an ad hoc study to evaluate the performances of the in vitro assays used would be useful as well.

## 5. Conclusions

Assessing the virulence potential of strains repeatedly isolated in the environment of an FPE is of interest since it can provide useful information to evaluate the consumer’s risk.

The persistent clusters studied belonged to different CCs, which presented different genetic characteristics from each other in terms of virulence.

This study provided for the first time data on the in vitro adhesiveness and invasiveness of CC218-ST2801 strains and added more data on the virulence characteristics of the CC1 and CC9 strains. The CC1 and CC218 strains presented the same accessory virulence genes, absent in CC9 isolates, with differences in allelic variants. However, if compared to CC1 strains, CC218 showed lower adhesiveness and invasiveness levels in vitro, presenting an ability to adhere and invade Caco-2 cells not significantly different from CC9 isolates.

Unexpected results were found for strains belonging to the same genetic cluster and presenting the same virulence gene profile, since they showed significant differences in the adhesion and invasion levels in vitro. However, to confirm this result, more strains belonging to the same cluster should be tested.

What we observed in this study confirmed that the ability of *Lm* to adhere to and invade human cells in vitro is not always decipherable from its virulence gene profile. However, no gene expression analysis was performed, and this could represent a future project to clarify the obtained data. Finally, to verify if there is correspondence between the in vitro adhesiveness and invasiveness of the studied strains and their effective virulence in a host, it could be interesting to extend the study using an in vivo model.

## Figures and Tables

**Table 1:** *Listeria monocytogenes* strains examined in this study: clonal complex (CC), cluster, sampling date, location, and environmental surfaces from which they have been isolated.

Strain ID	CC	Cluster (Cl)	Sampling Date	Sampling Area	Surface
Lm_2529	CC1	Cl_CC1	16 July 2020	Washing area	Floor grid
Lm_2604	CC1		16 September 2021	Cutting-processing room	Cold room—drain channel
Lm_2527	CC9	Cl_CC9	16 July 2020	Cutting-processing room	Drain channel
Lm_2603	CC9		16 September 2021	Cutting-processing room	Teflon table—legs and feet
Lm_2516	CC9		16 July 2020	Cutting-processing room	Sausage table—legs and feet
2021.TE.312882.1.40	CC9		13 May 2021	Washing area	Shoe–cleaning machine—brushes and external part
Lm_2513	CC218	Cl_CC218_A	16 July 2020	Cutting-processing room	Cold room—wall–floor connection
Lm_2525	CC218	Cl_CC218_B	16 July 2020	Cutting-processing room	Water puller
Lm_2568	CC218	Cl_CC218_C	13 May 2021	Cutting-processing room	Sausage table—legs and feet

**Table 2:** Comparisons of the virulence gene profiles among different clonal complexes (CCs).

Compared Clonal Complex	No. of Loci Having the Same Allele	Percentage of Equal Loci (n = 93)	% (95% C.I.)
CC218 vs. CC9	21	23%	15.3–32.1%
CC1 vs. CC9	21	23%	15.3–32.1%
CC1 vs. CC218	42	45%	35.4–55.3%

C.I.—confidence interval.

## Data Availability

The genome assemblies of the strains were deposited at DDBJ/ENA/GenBank under the BioProjects PRJNA961709 (Lm_2529, Lm_2604, Lm_2527, Lm_2603, Lm_2516, and 2021.TE.312882.1.40) and PRJNA821663 (Lm_2513, Lm_2525, and Lm_2568).

## Supplementary Materials

The following supporting information can be downloaded at: https://www.mdpi.com/article/10.3390/microorganisms11071659/s1, Table S1: Metadata of all nine *Listeria monocytogenes* strains used in this study. Table S2: Virulence loci detected in silico using the BIGSdb-*Lm* scheme. The numbers inside the cells represent the allelic variant of each locus. Blue: presence of the gene; red: gene with a mutation that leads to premature stop codons (PMSCs); and black: absence of the gene. Table S3: Virulence loci that are variable among the studied strains. Blue: presence of the gene; red: gene with a mutation that leads to premature stop codons (PMSCs); and black: absence of the gene.

## References

[1] EFSA, ECDC (2022). The European Union One Health 2021 Zoonoses Report. EFSA J..

[2] De Castro V., Escudero J., Rodriguez J., Muniozguren N., Uribarri J., Saez D., Vazquez J. (2012). Listeriosis outbreak caused by Latin-style fresh cheese, Bizkaia, Spain, August 2012. Eurosurveillance.

[3] Lomonaco S., Verghese B., Gerner-Smidt P., Tarr C., Gladney L., Joseph L., Katz L., Turnsek M., Frace M., Chen Y. (2013). Novel epidemic clones of Listeria monocytogenes, United States, 2011. Emerg. Infect. Dis..

[4] Makino S.-I., Kawamoto K., Takeshi K., Okada Y., Yamasaki M., Yamamoto S., Igimi S. (2005). An outbreak of food-borne listeriosis due to cheese in Japan, during 2001. Int. J. Food Microbiol..

[5] Smith A.M., Tau N.P., Smouse S.L., Allam M., Ismail A., Ramalwa N.R., Disenyeng B., Ngomane M., Thomas J. (2019). Outbreak of Listeria monocytogenes in South Africa, 2017–2018: Laboratory Activities and Experiences Associated with Whole-Genome Sequencing Analysis of Isolates. Foodborne Pathog. Dis..

[6] Kaptchouang Tchatchouang C.D., Fri J., De Santi M., Brandi G., Schiavano G.F., Amagliani G., Ateba C.N. (2020). Listeriosis Outbreak in South Africa: A Comparative Analysis with Previously Reported Cases Worldwide. Microorganisms.

[7] Gillesberg Lassen S., Ethelberg S., Björkman J.T., Jensen T., Sørensen G., Kvistholm Jensen A., Müller L., Nielsen E.M., Mølbak K. (2016). Two listeria outbreaks caused by smoked fish consumption-using whole-genome sequencing for outbreak investigations. Clin. Microbiol. Infect. Off. Publ. Eur. Soc. Clin. Microbiol. Infect. Dis..

[8] Lüth S., Halbedel S., Rosner B., Wilking H., Holzer A., Roedel A., Dieckmann R., Vincze S., Prager R., Flieger A. (2020). Backtracking and forward checking of human listeriosis clusters identified a multiclonal outbreak linked to Listeria monocytogenes in meat products of a single producer. Emerg. Microbes Infect..

[9] Halbedel S., Wilking H., Holzer A., Kleta S., Fischer M.A., Lüth S., Pietzka A., Huhulescu S., Lachmann R., Krings A. (2020). Large Nationwide Outbreak of Invasive Listeriosis Associated with Blood Sausage, Germany, 2018–2019. Emerg. Infect. Dis..

[10] Allen K.J., Wałecka-Zacharska E., Chen J.C., Katarzyna K.-P., Devlieghere F., Van Meervenne E., Osek J., Wieczorek K., Bania J. (2016). Listeria monocytogenes–An examination of food chain factors potentially contributing to antimicrobial resistance. Food Microbiol..

[11] Iwu C.D., Okoh A.I. (2020). Characterization of antibiogram fingerprints in Listeria monocytogenes recovered from irrigation water and agricultural soil samples. PLoS ONE.

[12] Radoshevich L., Cossart P. (2018). Listeria monocytogenes: Towards a complete picture of its physiology and pathogenesis. Nat. Rev. Microbiol..

[13] Ricci A., Allende A., Bolton D., Chemaly M., Davies R., Fernández Escámez P.S., Girones R., Herman L., Koutsoumanis K., EFSA_Panel_on_Biological_Hazards (2018). Listeria monocytogenes contamination of ready-to-eat foods and the risk for human health in the EU. EFSA J..

[14] EU-Reference_Laboratory_for_Listeria_monocytogenes, ANSES-Food_Safety_Laboratory (2023). EURL Lm GUIDANCE DOCUMENT to Evaluate the Competence of Laboratories Implementing Challenge Tests and Durability Studies Related to Listeria Monocytogenes in Ready-to-Eat Foods Version 3 10/02/2023.

[15] Bucur F.I., Grigore-Gurgu L., Crauwels P., Riedel C.U., Nicolau A.I. (2018). Resistance of Listeria monocytogenes to stress conditions encountered in food and food processing environments. Front Microbiol..

[16] Jordan K., Hunt K., Lourenco A., Pennone V. (2018). Listeria monocytogenes in the food processing environment. Curr. Clin. Microbiol. Rep..

[17] Orsi R.H., den Bakker H.C., Wiedmann M. (2011). Listeria monocytogenes lineages: Genomics, evolution, ecology, and phenotypic characteristics. Int. J. Med. Microbiol..

[18] Yin Y., Yao H., Doijad S., Kong S., Shen Y., Cai X., Tan W., Wang Y., Feng Y., Ling Z. (2019). A hybrid sub-lineage of Listeria monocytogenes comprising hypervirulent isolates. Nat. Commun..

[19] Doumith M., Buchrieser C., Glaser P., Jacquet C., Martin P. (2004). Differentiation of the major Listeria monocytogenes serovars by multiplex PCR. J. Clin. Microbiol..

[20] Ragon M., Wirth T., Hollandt F., Lavenir R., Lecuit M., Le Monnier A., Brisse S. (2008). A new perspective on Listeria monocytogenes evolution. PLoS Pathog..

[21] Vázquez-Boland J.A., Kuhn M., Berche P., Chakraborty T., Domínguez-Bernal G., Goebel W., González-Zorn B., Wehland J.r., Kreft J.r. (2001). Listeria pathogenesis and molecular virulence determinants. Clin. Microbiol. Rev..

[22] Maury M.M., Tsai Y.H., Charlier C., Touchon M., Chenal-Francisque V., Leclercq A., Criscuolo A., Gaultier C., Roussel S., Brisabois A. (2016). Uncovering Listeria monocytogenes hypervirulence by harnessing its biodiversity. Nat. Genet.

[23] Moura A., Criscuolo A., Pouseele H., Maury M.M., Leclercq A., Tarr C., Björkman J.T., Dallman T., Reimer A., Enouf V. (2016). Whole genome-based population biology and epidemiological surveillance of Listeria monocytogenes. Nat. Microbiol..

[24] Maury M.M., Bracq-Dieye H., Huang L., Vales G., Lavina M., Thouvenot P., Disson O., Leclercq A., Brisse S., Lecuit M. (2019). Hypervirulent Listeria monocytogenes clones’ adaption to mammalian gut accounts for their association with dairy products. Nat. Commun.

[25] Painset A., Björkman J.T., Kiil K., Guillier L., Mariet J.F., Félix B., Amar C., Rotariu O., Roussel S., Perez-Reche F. (2019). LiSEQ—Whole-genome sequencing of a cross-sectional survey of Listeria monocytogenes in ready-to-eat foods and human clinical cases in Europe. Microb. Genom..

[26] Shi D., Anwar T.M., Pan H., Chai W., Xu S., Yue M. (2021). Genomic determinants of pathogenicity and antimicrobial resistance for 60 global Listeria monocytogenes isolates responsible for invasive infections. Front Cell Infect. Microbiol..

[27] Félix B., Sevellec Y., Palma F., Douarre P.E., Felten A., Radomski N., Mallet L., Blanchard Y., Leroux A., Soumet C. (2022). A European-wide dataset to uncover adaptive traits of Listeria monocytogenes to diverse ecological niches. Sci. Data.

[28] Ireton K., Mortuza R., Gyanwali G.C., Gianfelice A., Hussain M. (2021). Role of internalin proteins in the pathogenesis of Listeria monocytogenes. Mol. Microbiol..

[29] Quereda J.J., Morel C., Lopez-Montero N., Ziveri J., Rolland S., Grenier T., Aulner N., Danckaert A., Charbit A., Enninga J. (2022). A Role for Taok2 in Listeria monocytogenes Vacuolar Escape. J. Infect. Dis..

[30] Quereda J.J., Meza-Torres J., Cossart P., Pizarro-Cerdá J. (2017). Listeriolysin S: A bacteriocin from epidemic Listeria monocytogenes strains that targets the gut microbiota. Gut Microbes.

[31] Loo K., Letchumanan V., Dhanoa A., Law J.W.-F., Pusparajah P., Goh B.-H., Ser H.-L., Wong S.H., Ab Mutalib N.-S., Chan K.-G. (2020). Exploring the pathogenesis, clinical characteristics and therapeutic regimens of Listeria monocytogenes. Microbiology.

[32] Disson O., Moura A., Lecuit M. (2021). Making sense of the biodiversity and virulence of Listeria monocytogenes. Trends Microbiol..

[33] Johansson J., Freitag N.E. (2019). Regulation of Listeria monocytogenes virulence. Microbiol. Spectr..

[34] Wiktorczyk-Kapischke N., Skowron K., Grudlewska-Buda K., Wałecka-Zacharska E., Korkus J., Gospodarek-Komkowska E. (2021). Adaptive response of Listeria monocytogenes to the stress factors in the food processing environment. Front Microbiol..

[35] Sibanda T., Buys E.M. (2022). Listeria monocytogenes Pathogenesis: The Role of Stress Adaptation. Microorganisms.

[36] Carpentier B., Barre L. (2012). Guidelines on Sampling the Food Pprocessing Area and Equipment for the Detection of Listeria Monocytogenes Version 3–20/08/2012.

[37] Guidi F., Lorenzetti C., Centorotola G., Torresi M., Camma C., Chiaverini A., Pomilio F., Blasi G. (2022). Atypical serogroup IVb-v1 of Listeria monocytogenes assigned to new ST2801, widely spread and persistent in the environment of a porkmeat producing plant of Central Italy. Front Microbiol..

[38] Moura A., Tourdjman M., Leclercq A., Hamelin E., Laurent E., Fredriksen N., Van Cauteren D., Bracq-Dieye H., Thouvenot P., Vales G. (2017). Real-Time Whole-Genome Sequencing for Surveillance of Listeria monocytogenes, France. Emerg. Infect. Dis..

[39] Portmann A.C., Fournier C., Gimonet J., Ngom-Bru C., Barretto C., Baert L. (2018). A Validation Approach of an End-to-End Whole Genome Sequencing Workflow for Source Tracking of Listeria monocytogenes and Salmonella enterica. Front Microbiol..

[40] Cito F., Di Pasquale A., Cammà C., Cito P. (2018). The Italian information system for the collection and analysis of complete genome sequence of pathogens isolated from animal, food and environment. Int. J. Infect. Dis..

[41] Bolger A.M., Lohse M., Usadel B. (2014). Trimmomatic: A flexible trimmer for Illumina sequence data. Bioinformatics.

[42] Wingett S.W., Andrews S. (2018). FastQ Screen: A tool for multi-genome mapping and quality control. F1000Res.

[43] Bankevich A., Nurk S., Antipov D., Gurevich A.A., Dvorkin M., Kulikov A.S., Lesin V.M., Nikolenko S.I., Pham S., Prjibelski A.D. (2012). SPAdes: A new genome assembly algorithm and its applications to single-cell sequencing. J. Comput. Biol..

[44] Gurevich A., Saveliev V., Vyahhi N., Tesler G. (2013). QUAST: Quality assessment tool for genome assemblies. Bioinformatics.

[45] Timme R.E., Wolfgang W.J., Balkey M., Venkata S.L.G., Randolph R., Allard M., Strain E. (2020). Optimizing open data to support one health: Best practices to ensure interoperability of genomic data from bacterial pathogens. One Health Outlook.

[46] Schiavano G.F., Ateba C.N., Petruzzelli A., Mele V., Amagliani G., Guidi F., De Santi M., Pomilio F., Blasi G., Gattuso A. (2022). Whole-genome sequencing characterization of virulence profiles of Listeria monocytogenes food and human isolates and in vitro adhesion/invasion assessment. Microorganisms.

[47] Wagner E., Fagerlund A., Thalguter S., Jensen M.R., Heir E., Møretrø T., Moen B., Langsrud S., Rychli K. (2022). Deciphering the virulence potential of Listeria monocytogenes in the Norwegian meat and salmon processing industry by combining whole genome sequencing and in vitro data. Int. J. Food Microbiol..

[48] Nightingale K.K., Windham K., Martin K.E., Yeung M., Wiedmann M. (2005). Select Listeria monocytogenes subtypes commonly found in foods carry distinct nonsense mutations in inlA, leading to expression of truncated and secreted internalin A, and are associated with a reduced invasion phenotype for human intestinal epithelial cells. Appl. Environ. Microbiol..

[49] Su X., Cao G., Zhang J., Pan H., Zhang D., Kuang D., Yang X., Xu X., Shi X., Meng J. (2019). Characterization of internalin genes in Listeria monocytogenes from food and humans, and their association with the invasion of Caco-2 cells. Gut Pathog..

[50] Cardenas-Alvarez M.X., Restrepo-Montoya D., Bergholz T.M. (2022). Genome-Wide Association Study of Listeria monocytogenes Isolates Causing Three Different Clinical Outcomes. Microorganisms.

[51] Raschle S., Stephan R., Stevens M.J.A., Cernela N., Zurfluh K., Muchaamba F., Nüesch-Inderbinen M. (2021). Environmental dissemination of pathogenic Listeria monocytogenes in flowing surface waters in Switzerland. Sci. Rep..

[52] Gou H., Liu Y., Shi W., Nan J., Wang C., Sun Y., Cao Q., Wei H., Song C., Tian C. (2022). The Characteristics and Function of Internalin G in Listeria monocytogenes. Pol. J. Microbiol..

[53] Bierne H., Sabet C., Personnic N., Cossart P. (2007). Internalins: A complex family of leucine-rich repeat-containing proteins in Listeria monocytogenes. Microbes Infect.

[54] Popowska M., Krawczyk-Balska A., Ostrowski R., Desvaux M. (2017). InlL from Listeria monocytogenes Is Involved in Biofilm Formation and Adhesion to Mucin. Front. Microbiol..

[55] Moura A., Disson O., Lavina M., Thouvenot P., Huang L., Leclercq A., Fredriksson-Ahomaa M., Eshwar A.K., Stephan R., Lecuit M. (2019). Atypical Hemolytic Listeria innocua Isolates Are Virulent, albeit Less than Listeria monocytogenes. Infect. Immun..

[56] Cotter P.D., Draper L.A., Lawton E.M., Daly K.M., Groeger D.S., Casey P.G., Ross R.P., Hill C. (2008). Listeriolysin S, a novel peptide haemolysin associated with a subset of lineage I Listeria monocytogenes. PLoS Pathog..

[57] Li F., Ye Q., Chen M., Zhang J., Xue L., Wang J., Wu S., Zeng H., Gu Q., Zhang Y. (2020). Multiplex PCR for the Identification of Pathogenic Listeria in Flammulina velutipes Plant Based on Novel Specific Targets Revealed by Pan-Genome Analysis. Front. Microbiol..

[58] Martinez-Rios V., Gkogka E., Dalgaard P. (2020). Predicting growth of Listeria monocytogenes at dynamic conditions during manufacturing, ripening and storage of cheeses—Evaluation and application of models. Food Microbiol..

[59] Tavares R.M., Silva D., Camargo A.C., Yamatogi R.S., Nero L.A. (2020). Interference of the acid stress on the expression of llsX by Listeria monocytogenes pathogenic island 3 (LIPI-3) variants. Food Res. Int..

